# On the Nature and Treatment of Stomach and Urinary Diseases, &c.

**Published:** 1841-04-01

**Authors:** 


On the Nature and Treatment of Stomach and Urinary Diseases: being an Inquiry into the Connexion of Diabetes, Calculus, and other Affections of the Kidney and Bladder, with Indigestion. By *William Prout*, M.D. F.R.S. Fellow of the Royal College of Physicians. Third Edition, much enlarged. London: John Churchill, 1841.

[Second Notice.]

The portion of Dr. Prout's able work which treats of the Diseases of the Stomach and Urinary Organs is divided into two books, the first treating of Functional Diseases, the second of Mechanical Diseases.

### Of Functional Diseases.

This Book is divided into Five Chapters, comprising the following subjects.

Chap. I. General observations on the Pathology of Aqueous assimilation and secretion.
Of an Excess and Deficiency of urine.

Chap. II. General observations on the Pathology of Saccharine assimilation and secretion.
*Section a.* Of Saccharine urine. *Diabetes.*
*b.* Of Oxalic acid; Oxalate of Lime.
*c.* Of Lactic acid.

Chap. III General observations on the Pathology of Albuminous assimilation and secretion.
*Section a.* Of an Excess and Deficiency of Urea.
*b.* Of Albuminous urine.
*c.* Of Lithic acid.
*d.* Of Cystic oxide.

Chap. IV. General observations on the Pathology of Oleaginous assimilation and secretion.
*Section a.* Of an Excess and Deficiency of fat.
*b.* Of Cholesterine and its deposits, &c.

Chap. V. General observations on the Pathology of the Incidental Mineral matters entering into the composition of organised bodies.

### I. General Observations on the Pathology of Aqueous Assimilation and Secretion.

Dr. Prout adopts the old distinctions of *urina potûs*, the urine voided after drinking, and moderate eating, which, however, he prefers calling the *urine of assimilation*—and *urina sanguinis*, that liberated from the blood, after the primary processes of assimilation have been completed.

Fluids, observes Dr. Prout, taken into an empty stomach usually soon find their way to the kidneys. If, therefore, for any purpose, we wish to produce a large flow of urine, we ought to administer fluids when the digestive processes are quiescent; as, by giving them at such times, we are not only more likely to effect our purpose, but avoid the risk of deranging the assimilating processes. The same remark applies to the employment of mineral waters, especially mineral waters of low powers, and requiring to be taken in large quantities. The same cautions with regard to the *time* of administration, are applicable to those mineral waters which are strongly impregnated with neutral saline matters, and which chiefly exert their effects on the bowels as purgatives. Such waters should be taken at periods when they are least likely to interfere with the assimilating processes; otherwise they often do more harm than good.

Farther on, Dr. Prout adverts to the circumstances that attend the appropriation of water. This is gradual. Thus the alimentary matters, during their *reduction* into chyle, are gradually and slowly combined with water; and that, on the other hand, the chyle, during its coversion into blood, is gradually *raised* or separated from its associated water. The supply of water, therefore, must be duly regulated.

"In the healthy condition of the stomach and of the system in general, when a *natural* thirst indicates the want of drinks; fluids taken into the stomach, even during the digestive processes, interfere with these processes much less than might be expected; for, by a beautiful provision, the superfluous portion of the fluids is removed from the stomach almost as fast as it is introduced. The fluids thus removed, however, are liable to be occasionally charged with unassimilated crudities, which, in subsequently passing through the kidneys, produce more or less derangement of these organs. On the other hand, when fluids are taken into the dyspeptic stomach during the digestive processes, they are apt to be retained; and after giving occasion to flatulent distension, and to many other annoying symptoms, they are either tardily taken into the system, and thrown on the kidneys loaded to excess with unassimilated matters; or they sometimes escape by the bowels; or are ejected by vomiting. Hence, as a general rule, the impropriety of drinking too much during a meal." 9.

The *urine of assimilation* is exceedingly variable in its properties. Generally it is more or less dark-coloured, above the average specific gravity, and becomes turbid on cooling, from a deposition of lithic acid; or from a mixture of lithic acid with various other matters, some of which have been taken into the stomach as food, and have passed through the system unchanged, more particularly in dyspeptics. The *urina sanguinis*, voided long after food has been taken, is the standard urine, and shews more particularly the condition of the kidneys and of the system in general. So, in examining the urine, two specimens are required; viz. the urine voided after a principal meal, as after dinner; and the urine voided in the morning before breakfast; from which two extremes we may generally obtain all the information that the urine is calculated to furnish.

Dr. Prout observes that *excess* or *deficiency of urine*, considered in reference to disease, mark opposite conditions of the system—the former constantly accompanying those complaints connected with a peculiar state of nervous irritability—the latter usually waiting on an inflammatory state of the system.

### II. General Observations on the Pathology of Saccharine Assimilation and Secretion.

Dr. Prout has long paid great attention to this class of disorders, and it still occupies his mind. He reminds the reader: *first,* that saccharine aliments cannot in their natural state form constituent principles of living animal bodies, but must previously undergo certain changes of a more radical character than either of the other primary alimentary principles. *Secondly,* that such conversion of the saccharine aliments is a distinct function, among the earliest to exist and among the last to be extinguished. *Thirdly,* that this function is twofold, primary and secondary. *Fourthly,* that the gelatinous tissues may be regarded as the representatives of the original saccharine aliments. These considerations afford a clue to the following points, which Dr. Prout treats of seriatim.

1. That both the primary and secondary assimilating processes are liable to be deranged, not only in degree, but in kind; and that the derangement of one of the classes of assimilating processes necessarily more or less involves the other. 2. That one of the derangements of the primary assimilating processes consists in the want of power to assimilate certain forms of the saccharine principle, (*e. g.* sugar;) while another derangement of the same processes consists in the imperfect or mal-assimilation of the saccharine principle, by which various unnatural bodies, *e. g.* oxalic acid, lactic acid, &c. are produced instead of albuminous matters. 3. That the derangements of the secondary assimilating processes resulting from, or connected with, the derangements of the primary assimilating processes above mentioned, consist in the imperfect development of the living animal tissues, and particularly of the gelatinous tissues; and that, during the further changes which such imperfectly developed tissues undergo, various unnatural and poisonous matters are generated, the nature of the greater part of which is unknown to us; though among them there appear to be occasionally included matters similar to, or identical with, those matters, developed during the primary assimilating derangements, viz. oxalic acid, lactic acid, &c. 4. And lastly, That the causes producing derangements, both of the primary and secondary assimilating functions, are partly innate, and consist in a peculiar predisposition to such derangements; and partly exciting, and consist in exposure to accidental circumstances to which all individuals are subject; such as depressing passions of the mind; a residence in certain unhealthy localities; long-continued errors in diet, &c.

1. It can easily be conceived that the primary assimilating functions may be disordered, and that their derangement will disturb the secondary. On this we need not dwell.

2. That the primary assimilating organs are, under some circumstances, unable to assimilate the saccharine principle is evident from the fact that, in diabetes, sugar has been repeatedly ascertained to exist in the sanguiferous system; a fact unequivocally demonstrating that the assimilating organs had failed to convert the saccharine aliment into the constituent principles of the blood.

In diabetes, says Dr. Prout, the function by which the saccharine principle is converted into the constituents of chyle, is suspended or destroyed. In other diseases, however, this important function is not destroyed, but *erroneously exerted;* and the consequence is, that many principles, some of them of a poisonous character, as, for instance, the oxalic acid, are developed from the saccharine principle during the assimilating processes. That oxalic acid *must* be occasionally developed in the system is evident from the fact, that this acid is found in the urine when it has not been taken into the stomach. Now, when we consider that oxalic acid, taken into the stomach, passes through the kidneys unchanged; and that those who take sugar in excess are often liable to oxalate of lime concretions; there can be little doubt that the oxalic acid found in the urine is occasionally developed in the primary assimilating organs, and most probably in the stomach itself.

3. There is reason to believe that, in the advanced stages of diabetes, sugar is developed during the secondary assimilation of the gelatinous, perhaps of the albuminous and oleaginous tissues. Nor can it be doubted that the converting function of the secondary assimilating processes is liable to be *erroneously exerted*, and to give birth to various unnatural products. Dr. Prout advances the following considerations to establish the probability of the oxalic and lactic acids being included among them.

"It will be shown hereafter, that the oxalic acid in small quantity occasionally passes through the system, without producing any remarkable external symptoms of its presence; it will be also shown, that in many other instances the presence of oxalic acid in the system is accompanied by certain cutaneous and other diseases more immediately connected with the gelatinous tissues. Now, although we cannot *prove* that the oxalic acid in the latter case is the result of the same derangements of the secondary assimilating processes which give immediate occasion to the cutaneous affection; yet when, in addition to the preceding facts, we further take into account the relation of the gelatinous to the saccharine principle; no supposition appears more probable, than that *one* of the derangements of the secondary assimilation of the gelatinous principle may consist in the development of oxalic acid. But if this reasoning be deemed inconclusive with respect to the development of the oxalic acid during certain derangements of the secondary assimilating processes; its validity, perhaps, will scarcely be questioned with respect to the development of the lactic acid during certain other derangements of the secondary assimilating processes; for on what other supposition can we explain the presence of those enormous quantities of lactic acid which occasionally exist in the system during rheumatic and hectic fever? Will any one contend that all this lactic acid is, in such instances, developed in the stomach?" 18.

Among the other unnatural principles occasionally developed from the saccharine, or from the gelatinous tissues, Dr. Prout thinks we may enumerate many matters generated in the miasmatic and contagious fevers of tropical climates, and even of our own. He refers to the *black vomit*, which is often powerfully *acid.* He thinks, too, that organic diseases in general, but particularly those of a deep-seated and malignant character, appear to be more frequently connected with derangement of the gelatinous, (*i. e.* of the saccharine,) than of either the albuminous or oleaginous radicals.

4. Dr. Prout makes some remarks on the *causes* of mal-assimilation of the saccharine principle.

The internal *predisposing* causes are generally innate or inherited. Previous attacks, too, predispose to fresh.

Among the external *exciting* causes, one of the most frequent is exposure to cold, and especially to *cold and moisture.* And Dr. Prout thinks that *malaria* has a good deal to do with diabetes. Perhaps the following observations may be considered as bordering a little on the fanciful :—

" Every one is acquainted with the familiar fact, that the most frequent and striking morbid appearances presented by the urine from slight causes, (such as a cold, indigestion, &c.,) are the common lateritious sediments. Now, the first circumstance that attracted my notice after the prevalence of the Asiatic cholera, was the disappearance of these sediments from the urine. The absence of these sediments was at first considered to be accidental ; but when, day after day, the same occurrence took place, I was induced to inquire attentively into the circumstance, with the view, if possible, of ascertaining the reason. On closer inspection, it was found that the urine of every individual examined, whether in apparent health or otherwise, not only presented the same absence of sediment ; but also assumed that peculiar appearance, which I had been accustomed to consider as characteristic of the presence of oxalic acid. As I had always previously found the oxalic acid diathesis of unusual occurrence in London, I was much struck with the phenomenon ; and on reflection it occurred to me, that it might be referable to the same unknown cause which was then producing cholera. I was led to this notion from the analogous effects above mentioned produced in the urine by malaria; and also by another curious fact, noticed below, which likewise took place about the time the Asiatic cholera first made its appearance, viz. a positive increase in the weight of atmospheric air, similar to what might be supposed to be produced by the diffusion of a heavy gaseous principle through the lower regions of the atmosphere. My conclusion therefore was, that the cause of the phenomenon in question, as well as of the cholera, was a poisonous body analogous to malaria, whose high specific gravity and feeble diffusive powers kept it near the earth's surface, along which it insensibly crept, particularly in low and damp situations. Whether this conclusion was legitimate or not, others must decide. I do not think the point worth contesting : but shall briefly mention the following additional facts, connected with the subject.

During the prevalence of the above condition of the urine, I likewise noticed, in almost every individual, an unusually acid state of the saliva, and of the cutaneous exhalations ; such as I had never, indeed, before noticed, except in the last stages of chronic diseases ; or in malarious affections. Besides these circumstances, I also saw, about the time the cholera prevailed, and a little afterwards, more cases of oxalate of lime renal calculi, and of formidable hæmorrhage from the kidneys, &c., than I had ever previously seen during the whole of the long period that urinary diseases had occupied my attention. As the cholera disappeared, the above state of things gradually subsided ; but I have sometimes imagined that the urine has never completely recovered its former condition. Indeed, during the last winter and spring, lithic acid deposites were comparatively rare in London ; and the urine assumed much the same appearance as during the prevalence of cholera, though in a less marked degree." 23.

At the period in question, people generally were particular in their diet, ate more animal food than usual, or, at all events, less vegetable food, and, probably, consumed more brandy and port. Whether this had any thing to say to the condition of their urine we leave to Dr. Prout to determine.

Among the remote causes of saccharine derangements, may be mentioned *diet.* A free consumption of sugar predisposes to saccharine diseases, as well as to the oxalic acid diathesis. So do the sorrel and rhubarb plants, and Dr. Prout reprobates the rhubarb puddings and pies of Spring. Of *tobacco* he speaks in the unkindest terms.

" Tobacco disorders the assimilating functions in general, but particularly, as

I believe, the assimilation of the Saccharine principle. I have never, indeed, been able to trace the development of oxalic acid to the use of tobacco; but that some analogous and equally poisonous principle (probably of an acid nature) is generated in certain individuals by its abuse, is evident from their cachectic looks; and from the dark, and often greenish yellow tint, of their blood. The severe and peculiar dyspeptic symptoms sometimes produced by inveterate snuff-taking are well known; and I have more than once seen such cases terminate fatally with malignant disease of the stomach and liver. Great smokers also, especially those who employ short pipes and cigars, are said to be liable to cancerous affections of the lips." 24.

We hope our smoke-producing youth will attend to this.

*Of Diabetes.*

Dr. Prout, to prevent confusion, proposes to restrain this term to affections in which the urine is *saccharine*—designating an excessive flow of urine unattended with sugar—*Diuresis.*

The specific gravity of diabetic urine has been stated to vary from 1·020 to 1·050. But Dr. Prout has once or twice seen it as low as 1·015 and often as high as 1·055, and higher. The quantity of urea is sometimes much diminished, though Dr. P. has never seen it absent; and, in some instances, urea is said to exist in diabetic urine in greater proportion than natural. Lithic acid also is usually found in saccharine urine in greater or less quantity; and in favourable cases of the disease, the quantity of this acid is often very considerable. The usual saline matters existing in the urine are met with in diabetic urine in nearly the same *relative* proportions as in health; but the *absolute* quantity of saline matters, viewed in relation to the quantity of urine passed, is much diminished. Sometimes diabetic urine contains a little blood; and not unfrequently albuminous matter, analogous to that of chyle.

Dr. Prout gives the following hint for ascertaining the period when the disease commenced.

"The commencement of diabetic attacks can seldom be accurately determined; but by inquiring minutely as to the period when the urine *was last observed to be turbid,* I have several times traced attacks very nearly to their origin. In such instances, patients have usually stated, that at some former period, the continued turbidity of the urine was such as to attract their observation; and on being questioned as to the supposed cause of such turbidity, some have ascribed it to exposure to cold; others to an attack of gout or rheumatism; others to disordered health from mental anxiety; &c. In most instances, the cessation of this turbidity was not accurately noticed; in a few, the termination was observed to take place rather abruptly; and the urine, on becoming clear, was likewise observed to become increased in quantity. Now it is probable that at the time the urine became clear, its saccharine condition commenced, or at least became confirmed; though, in general, the increased flow of urine was not so great, as to attract the patient's attention for several weeks; sometimes for several months, after this period." 29.

Dr. Prout gives a succinct account of the symptoms and progress of the malady; and comments on some of the former.

*Thirst* is worst in those who drink most. The skin is occasionally *moist.* There is, in some instances, inflammatory redness at the external orifice of the urethra; and sometimes phymosis.

*Emaciation* is not an absolutely constant symptom. Dr. Prout attended one patient who weighed twenty-three stone and a half, and another who weighed upwards of seventeen stone. He states that he knew a gentleman who long laboured under confirmed diabetes, and who recovered so far as to marry and to have two children; though the saccharine condition of the urine never left him. Indeed a saccharine condition of the urine exists in dyspeptic and gouty individuals much oftener than is supposed; and hundreds pass many years of their lives, with this symptom more or less constantly present, who are quite unaware of it, till the quantity of urine becomes increased.

*Terminations of Diabetes.*—" Phthisis, as already stated, is the most frequent. Besides phthisis, however, I have seen diabetes prove fatal by disease of the liver and jaundice; by apoplexy; by a peculiar affection of the stomach, brought on by improper food, or by over-distension; by acute gastritis induced by taking cold fluids when heated; by inflammatory fever excited by exposure to cold, and rapidly assuming the typhoid character; &c. Occasionally diabetes is said to terminate in incurable dropsy, and in various other affections. In short, a great many circumstances, which would not affect a sound constitution, often prove fatal in this disease. Hence a diabetic individual may be considered as existing on the brink of a precipice; and the general prognosis must be always unfavourable." 34.

*Causes.*—The lower animals seem to be exempt from diabetes—a curious, if a correct fact. A *predisposition* to the disease is more frequently inherited than acquired. Dr. P. has seen more cases of diabetes in individuals of the sanguine temperament with light or reddish hair, than in any other The disease, however, occurs in all temperaments; and perhaps next in frequency to the sanguine, in the melancholic temperament. In strumous individuals, with dark hair and eyes, fair skin, &c. diabetes often assumes its most unmanageable and fatal form. Diabetes is less frequent in women than in men; and rarely occurs in infancy or in old age, though there is a modification of it in infancy, described by Dr. Venables. Yet a predisposition may be acquired by a residence in a cold and damp situation, or in a malarious district, particularly if at the same time conjoined with a poor and unwholesome diet, or the too free use of sugar, &c.; also by venereal excesses; by the abuse of mercury; and, in short, by any cause having a tendency to sap the foundations of organic life; and more especially of the processes of assimilation.

The most frequent *exciting* causes that Dr. Prout has seen, have been exposure to cold; attacks of rheumatism and of gout; the drinking of cold fluids while heated; mental anxiety or distress arising from a variety of causes, such as a sudden reverse of fortune, &c. But others have blamed cider, injuries of the back, affections of the skin and cellular tissue.

" Were I permitted to draw a general inference from my experience, I should say, that diabetes usually *follows* cutaneous affections; and accompanies (perhaps *precedes*) the affections of the cellular tissue. Thus I have several times heard patients observe, that they were formerly subject to eruptions in varions parts of the body, but that such eruptions disappeared after the diabetic complaint became established; nor do I remember more than one instance, in which diabetes actually accompanied a severe cutaneous affection. On the contrary, diabetes very frequently, (as far as my personal experience goes,

*always*) accompanies carbuncles, and malignant boils or abscesses allied to carbuncles. This is a fact mentioned by several of the older writers; and is of great importance to surgeons, who usually have the management of these affections." 36.

Dr. P. mentions a case in point. He thus states the *proximate cause* of diabetes;—

"When *crystallised* sugar is taken into the stomach by a diabetic individual, it is reduced to a *low* state, remains more or less unassimilated, and passes through the system to the kidneys, by which organs (being already crystallisable) it is separated unchanged.

When *organised* saccharine principles, as farinaceous matters, &c. are taken into the diabetic stomach, they are in the first place reduced to the form of *low* sugar; part of which low sugar is assimilated as in the healthy stomach; while another part is modified or remains unassimilated. The portion that is assimilated is applied to the purposes of the economy: the portions modified and unassimilated pass together through the system to the kidneys, by which glands, the portion modified is disorganised, and finally appears in the urine as crystallisable sugar, along with the portion originally remaining unassimilated in the stomach. The same remarks are applicable to gelatinous, and in extreme cases, perhaps, to albuminous and oleaginous aliments. The *secondary* assimilating processes in diabetic individuals participate in the derangements of the primary processes just detailed;—that is to say, the gelatinous tissues are either reduced to sugar, and thus not assimilated at all; or they are imperfectly assimilated; or they are mal-assimilated; in all which conditions, the saccharine principle derived from the gelatinous and other tissues, may be supposed to pass through the system to the kidneys: by which organs, like similar matters brought from the stomach, the various modifications of the saccharine principle are further disorganised, and converted into crystallisable sugar."* 37.

Dr. Prout enumerates the favourable and unfavourable symptoms in diabetes. Among the *favourable* may be enumerated, a moderate flow of urine of a specific gravity not higher than 1035; the appearance in the urine of lithic acid either in its amorphous or crystallised form; the recent appearance of the disease, and absence of thirst; the retention or gain of flesh and strength; and more than all, immunity from organic disease, more especially from organic disease of the lungs. On the contrary, when the flow of urine is permanently excessive, and of high specific gravity; or when this secretion is pale coloured, opalescent, and serous; when the thirst, emaciation, and debility are extreme; or when organic disease, particularly of the lungs, is present, the chance of recovery is much diminished. But when, as is too frequently the case, several, or all of these unfavourable symptoms co-exist; the chance of recovery is not only diminished, but *absolutely hopeless.*

The *post-mortem* appearances in diabetes are too inconstant to permit us to consider them as more than concurrent or consequent affections. The most frequent appearances which Dr. P. has noticed have been rather of a

---

* "These views seem to be almost demonstrated by the important observations of Mr. M'Gregor, before alluded to. Mr. M'Gregor found that sugar is not only developed from vegetable and animal matters in the stomach; but that it exists in the blood, saliva, &c. and even in the alvine evacuations of a diabetic patient."

chemical character:—First, an enlarged, flaccid, and occasionally a congested state of the kidneys; a section of which organs, when first removed from the recently dead body, has usually assumed, on exposure to the air, a peculiar deep orange-red tint, difficult to be described; Secondly, a gorged condition of the veins terminating in the portal system, particularly of the veins of the mesentery; and an unusually dark-coloured and fluid condition of the venous blood throughout the assimilating organs; Thirdly, but not so constantly, a vascular state of the mucous membrane of the stomach, and upper portion of the alimentary canal.

*Treatment.*—The principle inculcated by Dr. Prout is this—Diabetes is a form of dyspepsia; and it ought always to be considered in a twofold light; as a simple saccharine condition of the urine, without any increase in its quantity, and as complicated, with a preternatural flow of that secretion. But, at present, the only means we possess for improving the quality of the urine are remedies that lessen its quantity.

*Diet* is the great thing. Dr. Prout does not approve of one exclusively animal, but considers a certain proportion of farinaceous matters proper. The ratio must vary with the assimilating powers of the patient. Of farinaceous matters, the *high* or *strong*, as the farina of wheat in the shape of bread, &c., seem to be most easily assimilated. The *low* kinds of farinaceous matters, as arrow-root, potatoes, &c., (with the exception perhaps of rice,) seem to be reduced to a species of sugar, more difficult of assimilation than the sugar from wheat-flour, &c., and in general, therefore, should be avoided. Every variety of the saccharine principle in its *crystallisable* form, is absolutely inadmissible as an article of food in diabetes. This rule excludes therefore, at once, all fruits, whether subacid or sweet; as well as every compound, natural or artificial, into which sugar enters. Dr. P. has known a few pears undo, in two or three hours, all that had been gained in months. Allow no latitude to patients. Like Dr. Johnson they can abstain, when they cannot be abstemious.

And *quantity* should be attended to. As a general rule with respect to diet, it may be said that a quantity, greater or less according to circumstances, but always strictly regulated, should be taken at periods of four, five, or six hours; and that at the time of taking solid food, and for an hour or two afterwards, all fluids should be abstained from as much as possible. Dr. P. would say that mutton or beef, plainly cooked, and particularly mutton-chops or beef-steaks, rarely done, should be taken twice in the twenty-four hours; and that the other meals should consist of any simple article that can be prepared from farinaceous matters with milk, eggs, &c. only. Sometimes oleaginous matters, more particularly butter, agree well. When the reducing function is impaired, as happens in a few instances, a system of diet less solid, and consisting of animal matters reduced to the pulpy state by stewing after the French fashion, will be more appropriate.

The management of drinks is as important as that of solids. A certain indulgence must be allowed.

"The Bristol Hotwell, and other waters containing carbonate of lime in solution, have been long celebrated in diabetic affections; and, as Dr. Marsh observes, they appear to quench the thirst in those affections better than most other mere diluents. Allied to these are waters artificially impregnated with lime or magnesia; as lime-water, which, either alone, or with milk, has been a favourite remedy in diabetes with some writers; the same may be said of water containing magnesia held in solution by carbonic acid, &c. Waters containing fixed alkalis, and their salts, are generally too diuretic in their effects to be recommended in this affection; yet when saturated with carbonic acid gas, and held in the mouth for a few seconds without swallowing them, they often remove or mitigate thirst better than most other fluids. All the stronger saline waters, from their diuretic proportions, should be carefully avoided. As a simple diluent, I am disposed to think very highly of distilled water. The use of water, however, in all its forms, should be sparingly allowed, as it is exceedingly liable to be abused; and various animal decoctions, milk, &c.; should be taken instead. When the patient has been in the habit of taking fermented liquors, I have been accustomed for some years past to recommend sound porter in preference to wine or spirits. The *quantity* must be determined by the circumstances of the patient; but the minimum quantity should be rarely surpassed. With very few exceptions, I have seen more relief from thirst, and more support given by porter in diabetic cases, than by any other means whatever. As general rules, also connected with the subject of fluids, it may be observed; first, that as the sudden abstraction of fluids in diabetic cases is sometimes followed by unpleasant consequences, the quantity should be *gradually* diminished; secondly, that in order to induce the patient, whose craving is generally after *cold* drinks, to take as little as possible, all fluids should be recommended in a *tepid* state; and lastly, that fluids should be taken at those periods in preference to others, when the stomach is not loaded with solid food." 45.

The *remedial treatment* must be conducted on general principles. Blood-letting is useful at the commencement. *Purgatives* Dr. P. has only found of use, as simply regulating the bowels, and he excludes the saline class, with the exception of phosphate of soda. *Sudorifics* are important.

Dr. Prout speaks highly of *opium.* Yet he believes that they who have stated that it *cures*, have over-praised it. Dover's powder is usually the best form, but it may agree worse than other preparations.

"Sedatives are often advantageously combined with astringents and tonics in the chronic forms of diabetes. Thus, opium may be associated with tannin, or with its modifications, catechu and kino; also with the mineral acids, and particularly with the sulphuric acid, either alone, or in combination with quinine, iron, zinc, copper or alumine. I have occasionally had recourse to all these combinations; but in general have preferred the supersulphate of quinine or of iron; and at the same time made it the rule to do with as little opium as possible. The blue phosphate, and the carbonate of iron, are also excellent remedies. Of the phosphate of iron in particular I am disposed to think very favourably; but I have been disappointed with the use of phosphoric acid; which has not in my hands produced the good effects some have ascribed to it; even when very freely and perseveringly administered." 48.

When diabetes is complicated with other diseases, *they* must be considered in *its* treatment. This is necessary more particularly in its early stage. Such a complication is frequently hepatic disorder or disease, and this leads our author to make some observations on *mercury.* He deprecates its abuse—the indiscriminate and destructive calomel and black-draught system, which, though in its glory twenty years ago, is by no means obsolete now. Dr. Prout lays down these maxims for its use.

First, Mercury ought in no instance to be administered for those slight deviations from health which can be readily removed by safer expedients.

Secondly, Mercury ought to be cautiously administered to strangers; and to those on whose constitution its effects have not yet been ascertained.

In the following observations we perfectly concur, and, indeed, we are in the habit of putting the case in this very manner to our own patients. We wish all medical men and the public would take the same rational views.

"The stimulating effects of mercury may be analogically illustrated by the stimulating effects of dram-drinking. As the stomach accustomed to ardent spirits will scarcely tolerate any weaker beverage; so the liver, accustomed to the stimulus of mercury, will hardly respond to any other influence. Those, therefore, who in early life have on all trivial occasions resorted to the powerful stimulus of mercury, like early dram-drinkers, are usually obliged to persist in the baneful habit. The truth of this analogy will be scarcely questioned: for the most superficial observer must have noticed, that patients who habitually take calomel are more than ordinarily subject to periodical congestions, or *biliary* attacks as they are termed; and that such biliary attacks will rarely yield to any other remedy than calomel. Nor is the insensibility to gentler expedients, thus too often produced in the soundest constitutions by the use of mercury, its only fault; the habitual use of this remedy is capable of exerting positive mischief on the assimilating functions and on the kidneys of some individuals; as will be shown in subsequent parts of this volume. Moreover, those who are under the influence of mercury in a degree far short of salivation, are notoriously liable to take cold, rheumatism, &c., from slight exposure; and various formidable and fatal diseases, as phthisis, &c., can be often distinctly traced to such exposure under the influence of mercury." 51.

Mercury is a very proper medicine when there is a disease for which it is appropriate, to combat, and the patient's constitution is adapted for it. In many chronic diseases mercury is beneficial, but not in the ratio of the largeness of the dose. Our author has never seen mercury do good in diabetes; almost invariably it has been mischievous. This mischief has been displayed in various ways connected with the urinary secretion; that is to say, the specific gravity of the urine has been increased; or the secretion has become serous, or otherwise deteriorated. Moreover, when the effects of the mercury have ceased, the patient has usually become worse than before; and the disease, after assuming its most unfavourable form, has rapidly advanced to its fatal termination. Nay, when the disease has been partially got under, he has seen a few grains of blue-pill inadvertently given, in the short space of a day or two, double and even triple the quantity of urine; and thus the benefit, to obtain which perhaps months had been required, has been lost as it were in a moment, and the patient has been reduced to a worse state than he was in at first.

Dr. Prout is inclined to limit the use of mercury in diabetes to the following cases:—First, general inflammatory or phlogistic fever; Secondly, acute or chronic inflammation of the liver; and Thirdly, the temporary congestion apt to occur in those individuals, who have been long accustomed to the stimulus of mercury.

1. Acute inflammation is rare in diabetes. When such does follow exposure to cold, the acute stage is of brief duration and rapidly passes into the adynamic form, with a disposition in the inflamed parts to become gangrenous. The acute stage of such attacks therefore is so transient, that it usually disappears before medical advice can be obtained; otherwise, if promptly met at the very beginning by free abstraction of blood, and the judicious application of calomel and opium, there is a chance that the progress of such attacks may be arrested. But if the peculiar adynamic state become once established, no treatment seems to avail.

2. Our author has seen chronic inflammation of the liver with congestive enlargement and jaundice, and too frequently organic disease, accompany diabetes. In the treatment of complications of this nature, the rule to be attended to, is to do everything in the first place that can be done by the aid of other expedients; so as to leave that only to be done by mercury, which mercury alone will accomplish. General and local activity, therefore, should be reduced as speedily and effectually as possible, by the abstraction of blood, by blistering, and by other well-known expedients; and when these means have effected all they are capable of doing, the aid of mercury may be resorted to. The peculiar circumstances of the case must, in some degree, determine the mode of employing this active remedy; but in general as little as possible should be given internally, and the form of inunction, plasters, &c., should be preferred. When given internally, mercury should, for the most part, be conjoined with opium; and of the different preparations of the drug, perhaps calomel so associated constitutes, on the whole, the least objectionable mode of administration.

3. The occasional use of alterative doses of mercury, either combined with sedatives or with mild purgatives, or with both, according to circumstances, is beneficial in some constitutions; and particularly in those who have always from early life been previously accustomed to the stimulus of mercury.

The principles of treatment, which have been detailed, being adhered to, Dr. Prout has seen a few cases in which the saccharine quality of the urine has entirely disappeared; and a very great number of cases, in which the symptoms have been so far subdued as to give little trouble to the patient. But the latter must consider himself through life an invalid, and submit to the restrictions and employ the care which such a state demands.

### *Diabetic Diuresis in Young Children.*

In young children, says Dr. Prout, as in adults, diuresis is a symptom of very different forms of disease; in all these diseases the urine, as well as being excessive in quantity, is more or less unnatural. Thus in infantile diuresis the urine almost always contains albuminous matters. In other instances an excess of the urea, or of the phosphates, is present; while in a few cases saccharine matters, more or less perfectly developed, exist either alone, or in conjunction with the above or other unnatural ingredients.

"The saccharine diuresis of young children usually commences soon after the period of weaning. From having been up to that time healthy, the child begins to get dull and inactive, and to daily lose flesh. The skin also becomes harsh and dry, and feels hotter than natural. As the disease proceeds, the bowels become irregular, and the motions assume an unnatural, often greenish appearance; the abdomen also usually becomes prominent, so as to lead to the suspicion of mesenteric disease. The pulse is quick, and denotes great irritability. In connexion with these symptoms, the quantity of urine begins to gradually increase, at first so slowly as to escape notice; but at length the quantity becomes so great, and the accompanying thirst so urgent, that these circumstances can no longer be overlooked. The urine is sometimes quite limpid; at other times of a pale straw or greenish colour; sometimes opalescent or milky. The specific gravity fluctuates considerably even in the same individual; and though it often falls within the diabetic range, the specific gravity seldom reaches the high point of the diabetic urine of adults. From the almost invariable presence of albuminous matter more or less perfectly developed, and which acts as a ferment, the diabetic urine of children is apt to undergo rapid changes from saccharine or acetous fermentation, or from both; and soon begins to emit an odour somewhat resembling sour milk.

This disease occurs most frequently in the children of profligate, dyspeptic, and gouty individuals, more especially in large towns; while the immediate exciting causes in such predisposed individuals are commonly want of air and proper nourishment; or injudicious management. The disease is of a formidable nature, and generally proves fatal; particularly if its nature be overlooked at the outset; and it be in consequence improperly treated. The treatment consists, in the first place, in removal to a purer air, or to the sea; and in the employment of a regulated and nutritious diet, consisting, as far as the tender age of the little patient will admit, of animal matters; at least, sugar and all sweet articles should be avoided. The state of the bowels should be attended to; and while calomel purges should be most carefully shunned, some gentle alterative, as the *hydrarg. cum creta,* combined with rhubarb and magnesia; or the carbonate of soda, calumba, &c. may be often given with advantage. The warm sea bath, with friction upon the skin, &c., will be also useful. The quantity of fluid taken should be strictly limited; and in addition to the other means, such tonics as appear to be suited to the age and circumstances of the patient, may be given with advantage. Dr. Venables, who first drew attention to this disease in children, recommends the use of the blue phosphate of iron; and this or the carbonate of iron, combined with a little magnesia or calumba, is often highly useful." 58.

### *Of the Oxalic Acid Diathesis.*

When oxalic acid is produced in the system, the urine is generally transparent, and remarkably free from sediments; of a pale citron-yellow, or greenish, hue; and of moderate specific gravity; that is to say, the specific gravity usually oscillates about 1020 as a mean point, but is often less than this—a circumstance chiefly referable to variations in the quantity of the urine secreted; which is frequently above the healthy standard.

These properties of the urine may induce suspicion, but judgment must not be definitely pronounced on them.

The symptoms are rather of the irritable or nervous class, than of the congestive or inflammatory. Dyspepsia, attended with flatulency, is complained of, and both the bowels and the biliary secretion are capricious and irregular. In individuals of the sanguine temperament, particularly when subject to cutaneous diseases, the constitutional symptoms are usually manifested in the form of extreme irritability of temper or manner; more especially if the cutaneous affection has, from any cause, been suddenly repelled. In individuals of the melancholic temperament, on the contrary, the constitutional symptoms usually partake of the desponding and hypochondriacal character.

When the obvious presence of a small calculus in the kidney or bladder leaves the nature of the case no longer doubtful, and occasions for the first time the appearance of blood in the urine; the occurrence, whether accompanied by pain or not, is apt to forcibly attract the patient's attention. He forgets all else. A nephritic attack occurs, his troubles pass away with his calculus, and he recovers, for a longer or a shorter period, his former state of health.

The oxalic acid diathesis is sometimes associated with serous urine, and with organic disease of the kidney, particularly in young subjects; in which case the urine is generally opalescent and of a greenish tint. When a great deal of saccharine matter is consumed as food, the urine is often of considerable specific gravity, and contains sugar as well as oxalic acid. Moreover, when sugar, and particularly oxalic acid, are thus freely taken, oxalic acid may be frequently detected in the urine; and the sediments deposited almost always contain more or less of oxalate of lime.

Sometimes the symptoms are very slight—sometimes they are prominent. Hæmorrhage from the kidneys is perhaps more frequently produced by oxalate of lime, than by any other form of concretion. This may depend in part on the peculiar form of the calculus; but the chief cause probably lies in the nature of the diathesis. But amongst the hundreds who suffer from this, a few only suffer from calculus.

*Causes.*—Hereditary predisposition is not so common as in diabetes. Syphilis is reproached by Dr. Prout as one cause. The oxalic acid diathesis occurs in all temperaments; but individuals of the sanguine temperament on the one hand, and of the melancholic on the other, seem to be most liable to it. When the diathesis is strongly marked, the skin in all temperaments is apt to assume an unnatural appearance difficult to describe, but the *colour* of which may be said to vary from dull greenish yellow in the sanguine, to dark olive or livid in the melancholic temperament. Both classes of individuals also are often liable to boils, which in old and enfeebled habits are apt to degenerate into carbuncles.

The periods of life most subject to it seem to be between two and twenty-four, and forty and sixty-five years of age.

The most effective of the *exciting* causes of the oxalic acid diathesis is a residence in a damp and malarious district. But it is not brought on by mere exposure to cold, nor by rheumatism, nor gout. It often, however, accompanies chronic rheumatism, and occasionally follows gout. The abuse of sugar has frequently produced it—so have rhubarb tarts, &c.—the depressing passions will give birth to it—cutaneous affections frequently attend it.

*Prognosis.*—In slighter cases there is no affection more manageable, if properly treated. In severe cases, particularly if complicated with organic disease, there is no affection more formidable, nor more apt to take on a malignant and intractable character.

*Post-mortem Appearances.*—In the few instances in which Dr. Prout has been enabled to examine the bodies of persons who have died with this diathesis, the immediate cause of death has been either organic disease of the kidneys, (generally combined with oxalate of lime calculus,) or some malignant disease of other organs. As in diabetes, there has been great tendency to acidity in the system; and the veins of the abdominal system have been unusually congested with dark-coloured blood.

*Treatment.*—The diet must nearly resemble that prescribed for diabetes. The patient should abstain from all saccharine articles of food, and his diet should consist principally of animal, and of the stronger farinaceous matters.

" As, however, the *reducing* function of the stomach is often considerably impaired in this diathesis, solid and indigestible matters should be sparingly taken, or shunned altogether. Hence the French cookery, by which animal and other matters are reduced to a semifluid or pultaceous mass, often agrees better than the crude and solid chops or steaks of this country. There are many exceptions, however, to this observation; and if the reducing function be not very much impaired, it is proper in all instances to take a certain portion of food of an easily reducible character; the best method of restoring the reducing, as well as all other weakened functions, being to moderately exercise them. When the stomach, as is often the case, cannot reduce oleaginous aliments, butter should be avoided; otherwise there is no objection to its use. As *drinks,* fermented liquors should in general be abstained from as much as possible; in this respect, however, everything will depend on the previous habits of the patient. Sometimes a little good porter agrees well, and may be taken. When porter is deemed objectionable, weak brandy and water is preferable to most wines; particularly those wines containing unfermented sugar. Sound and dry sherry, or even hock and claret, occasionally agree, and may be cautiously taken in some cases. The quality of the water employed is of the utmost importance. Those whose assimilating organs form oxalic acid, and who at the same time drink water containing lime in solution, are exceedingly liable to get an oxalate of lime calculus. The purest water, therefore, that can be obtained, even distilled water, should in all instances be preferred." 68.

The fixed alkalis are seldom beneficial, particularly in large doses; in which form they often do absolute mischief. The volatile alkalis, combined with camphor and sedatives, in cases of great irritability, are sometimes useful. The mineral acids, either alone or combined with tonics, as the sulphate of iron or of quinine, are usually grateful to the stomach, and may be taken with advantage. But when the mineral acids begin to occasion a deposition of the lithate of ammonia or the lithic acid, their exhibition must be suspended. Indeed, in all instances, the mineral acids require to be left off after a time; as, when too long persisted in, they not only cease to do good, but in most instances do harm. Where the patient lives in the country, Dr. Prout commonly recommends the use of the muriatic acid, (or nitro-muriatic acid, as the case may be,) to be persisted in till the lithate of ammonia, or the lithic acid, begins to appear in the urine; or for *a month;* and by adopting such a course of acids three or four times in the year, and by a carefully regulated diet, he has seen the diathesis removed. The hydrocyanic acid, with or without digitalis, is often serviceable in relieving the flatulence and palpitation of the heart. Nearly the same remarks apply to the use of mercury in this diathesis as in diabetes.

### Of Lactic Acid, &c.

The prominent features of these derangements are more boldly marked in tropical climates than in this country, and Dr. Prout merely presents a sketch of them at present. Along with lactic acid, and the nearly related acetic acid, he considers the subject of the muriatic and other acid principles usually developed at the same time.

*Of the development or presence of the Lactic Acid, &c , during the primary assimilating processes.*—The lactic and muriatic acids are principally derived from the blood, and from the matters secreted or introduced into the stomach; while the oxalic, butyric, acetic, carbonic, and perhaps occasionally the lactic, acids, are developed from the food during its imperfect assimilation; which imperfect assimilation is often a concomitant circumstance attending the abnormal development of the lactic and muriatic acids. When such abnormal development occurs, though both acids may be in excess, one usually predominates. Dr. Prout thinks the following observations may afford a clue to the reason of the predominance of the one acid or the other. That of the muriatic acid seems in general to denote a phlogistic or inflammatory state of the system; while the predominance of the lactic acid marks rather a state of irritation. This is the general law, though there are many exceptions to it. Dr. P. has commonly found in the dyspepsia of plethoric gouty individuals "the predominating acid the muriatic acid; so also in what are called *bilious attacks,* and gall-stones, as they occur in the same class of individuals, the predominating acid is usually the muriatic. In these and similar instances the stomach may be primarily in fault; but, in general, the stomach is affected by sympathy with some distant part. Thus, in *bilious* attacks, the hepatic system is supposed to be congested, so as to perform its functions imperfectly; or, as happens in some severe cases, the fault lies in one of the great nervous centres. So, in the same class of subjects, gouty or inflammatory action of the kidney, uterus, &c., is apt to be accompanied by a predominance of the muriatic acid. On the other hand, the same derangements and remote sympathies, when they occur in weak and delicate, or in *nervous* subjects, are very often attended by the presence of an excess of lactic acid in the stomach. Moreover, in *all* dyspeptic subjects, hard and crude indigestible matters, when taken into the stomach, irritate that organ, and cause it to throw out a large quantity of the mixed acids, in which the lactic acid almost always predominates; especially in the weak and delicate. An excess of acid, and particularly of lactic acid, in the stomach, is frequently accompanied by more or less of gastrodynia; that is to say, of rheumatic neuralgia, similar to that affecting other nerves of sensation. This happens most frequently in gouty and rheumatic subjects, in whom the exciting cause of the acid development has been some foreign indigestible substance.

With respect to the other acids formed in the stomach, these seem also to occur most generally in dyspeptic individuals in whom the muriatic and lactic acids abound; and in whom, in consequence, the digestive processes are imperfectly performed. These acids appear to be chiefly derived from the food, and therefore are probably various in their nature. Among others, the carbonic acid is frequently developed not only from the food, but apparently from the stomach itself; and, in its gaseous form, occasionally proves a source of flatulent eructation. Another, and by far the most troublesome, source of flatulence, is azote. This, in nervous subjects, is occasionally developed from the stomach in enormous quantities in conjunction with the lactic, and particularly with the oxalic acid, as formerly mentioned.

At other times azote is probably derived from the food; but from whatever source this gaseous principle be derived, it usually gives much annoyance; for while the carbonic acid gas, on account of its stimulating qualities, generally escapes from the stomach, the passive character of the azote, and the peculiar spasmodic constriction which usually accompanies its development, cause it to be retained; and thus, by distending the stomach, to add greatly to the miseries of the patient."

The muriatic acid, if formed from the muriate of soda in the blood, may be neutralized by the soda set free by the liver. But if there be lactic acid in excess, there is no alkali to neutralise it. The consequence is, that the free acid is either taken up with the chyle into the lacteals; or descends into the intestines; where, in conjunction with other acids there developed or separated, it produces various *secondary* symptoms. These symptoms are either local or remote; and moreover differ remarkably in different individuals, and at different ages. In adults, while the acid ingesta remain in the duodenum, great discomfort and uneasiness of various kinds are experienced. Again, peculiar symptoms, among which are a sense of heat and painful colic, often attend the passage of acid matters down the small intestines. If the acid matters pass unneutralised from the cœcum, they usually give occasion to more or less of pain throughout the region of the colon, and sometimes excite diarrhœa. In young children, these and many other distressing symptoms, produced by acidity in the primæ viæ, are still more strongly marked. Thus acid matters, in passing from the duodenum through the small intestines, often produce violent tormina, occasionally terminating in intus-susception; while the presence and retention of acids in the cœcum and colon not unfrequently give occasion to convulsions.

These symptoms may be occasioned by the absorption of acids into the system, as well as by their presence in the primæ viæ. The following account of the effects of *Acidity in the Cœcum*, will, we apprehend, be new to our readers:

"Excessive acidity of the cœcum is generally accompanied by a deficient secretion of bile; and sometimes by a complete temporary suppression of the bilious discharge, apparently from spasmodic constriction of the common gall-duct; or, it may be, of the biliary ducts themselves. In this state of things, all individuals feel more or less of uneasiness; but the point we wish to mention is, that certain individuals under these circumstances experience what is called nervous headache. This species of headache is frequently accompanied by nausea; is confined to the forehead; and when severe, produces complete intolerance of light and sounds, and a state of mind bordering on delirium. After a greater or less period the pain ceases; sometimes quite suddenly; and the remarkable circumstances to be mentioned are, that this sudden termination is preceded by a peculiar sensation (sometimes accompanied by an audible clicking noise) in the region of the gall-ducts; that immediately afterwards, a gurgling sensation is felt in the upper bowels, as if a fluid was passing through them; and that in a few seconds, when this fluid, which we suppose to be bile, has reached the cœcum, the headache at once vanishes like a dream. One of the greatest martyrs to this species of headache I have ever seen, invariably experiences the train of symptoms above described; and I have witnessed it in a greater or less degree in many instances; indeed I have experienced it in my own person." 75.

*Of the Development of the Lactic Acid, &c. during the Secondary Assimilating Processes.*—The effects of the acids developed during the secondary assimilating processes are with difficulty distinguished from those already described.

In dyspeptic persons who suffer from acidity in the primæ viæ, the secondary assimilating processes are deranged. The symptoms have more or less of a periodic character, and show themselves in occasional attacks of bilious congestion, gout, lithic acid gravel, catarrhal affections, ague, rheumatism, &c., according as exposure to cold, malarious influence, &c., co-operates with the original predispositions, and determines their nature.

Those who suffer least from derangements of the primary digestive processes, often experience the greatest inconvenience from the derangements of the secondary class, or from their consequences. They boast that nothing disagrees with them, and eat of every thing that comes in their way. In the prime of life, and in sound constitutions, this state of things goes on for periods varying according to circumstances, and particularly according as individuals are indolent or active. In almost all instances, however, sooner or later, the urine becomes loaded, the liver congested, and more or less of fever and derangement of the stomach and bowels,—in short, what is usually called a *bilious* attack, takes place. A calomel pill and black draught seem to set matters right, and the same habits and same round of changes recur.

"There are some individuals in whom, though the primary assimilating processes are imperfectly performed, and though they eat and drink immoderately, and of everything that comes in their way, suffer comparatively little inconvenience from their excesses; nay, even seem to be all the better for them, if we believe their own account of the matter. In such individuals the bowels are usually lax, and enormous quantities of fæces are passed, consisting of matters taken as food, and which have never been assimilated at all; while the portion that has been imperfectly assimilated and taken into the system readily passes off by the kidneys, skin, &c., without materially affecting the constitution. Subjects of this description, for the most part, are of a lax scrofulous habit, and require to be well supported, in order that enough of matters may be assimilated by their imperfect organs to carry on the vital processes. If such individuals be well fed, they often attain old age; but they are liable to hypertrophies and morbid growths of various kinds; and generally die of dropsy connected with extensive organic disease. The children of such individuals, if they have any, which is frequently not the case, are usually sickly, and very often die in their infancy; and a third generation of such a race, unless counteracted by favourable intermarriages, rarely exists. Habits of this description are met with in various grades, and states of combination; and individuals in whom such habit is associated with gout, gravel, or in short, with any other inherited predisposition to disease, are commonly remarkable sufferers." 78.

Dr. Prout reverts to the circumstances that, in full livers, of a healthy constitution, usually precede a "*bilious attack.*" When acid and unassimilated matters accumulate, as they do in the dyspeptic, they are thrown off periodically by the bowels, or by other organs. In the strong, the symptoms take the form of simple feverish excitement, with more than usual derangement of the stomach and bowels, and generally sickness and diarrhœa; in the delicate, the weak part, whatever it may be, is involved more particularly under the influence of cold. Thus every one must have observed that when the system is so charged, he is liable, on the slightest exposure, to get cold; particularly if the lungs are in the least degree predisposed. Others, as above observed, in such a state of the system from a similar exposure, get an attack of rheumatism; others gout or erysipelas; others a nephritic attack. Bilious persons, too, in such a state, on comparatively slight exposure to malarious influence, get an attack of rheumatism or ague. Dr. Prout adds:—

"When a cold is caught, particularly in old and dyspeptic individuals, one of the first symptoms often experienced is an immense discharge of glairy aqueous fluid from the salivary glands, and even from the stomach, (analogous to the *water-brash,*) and which is not acid. This discharge of fluid is often accompanied by indigestion and flatulence, and a sort of spasmodic constriction of the cardia, so that the gaseous matters are expelled with difficulty. The watery discharge has often a *cold* feel, and is frequently most copious in the night. The stomach also feels *cold.* These phenomena seem to occur most frequently in gouty and rheumatic subjects, and in some are constantly present, in a greater or less degree; but in all are increased by exposure to a damp and raw atmosphere. Under these circumstances, the stomach is apt to be particularly embarrassed by any indigestible and cold articles of food, which aggravate the affection. This state of the salivary glands, &c., seems to resemble closely that state of the skin which gives occasion to what is termed a *cold sweat;* or that condition of the kidneys produced by exposure to cold, which in certain habits is accompanied by diuresis, &c. Such a state is always attended by a peculiar atonic condition of the nerves of the parts affected; which nervous atony paralyses or renders the organs insensible, as it were, to every stimulus except that of water, which in consequence passes off in excess. In aged individuals who are constantly subject to this flow of watery fluid in a profuse degree, the discharge seems to operate vicariously to the kidneys, and perhaps to other organs; and I have several times seen coma supervene on its sudden cessation." 79.

Dr. Prout would infer, from the facts already stated, that the severe derangements of the secondary assimilating processes going on all over the system, are nearly allied to certain forms of *fever;* while the local and specific derangements are identical with certain specific *inflammations.*

He continues:—

"We may perhaps be allowed to assume, without opposition, that *some* diseases to which we apply the terms *fever* and *inflammation,* are, *practically* speaking, at least, what we have above inferred them to be, viz. only severer derangements of the secondary assimilating processes, modified by the peculiar nature of the organs or textures in which such derangements exist—inferences that will enable us to explain the principles on which derangements of the primary assimilating processes predispose to the peculiar derangements of the secondary processes now under consideration; and which we consider to be nearly connected, if not identical, with those forms of fever and inflammation usually denominated *intermittent fevers, rheumatism,* and *neuralgia.*" 80.

The exciting cause of those diseases is generally admitted to be malaria. Yet every one who has observed these diseases attentively, will probably admit, that the derangements of the assimilating organs constitute one of the first perceptible links in the series of symptoms; and, moreover, that these derangements of the assimilating organs are usually accompanied by the presence of great acidity in all parts of the system. Thus, in ague and rheumatism, during the sweating stages of the paroxysms, immense quantities of acid (chiefly of lactic acid) are thrown off by the skin; and sometimes by the kidneys. In these cases the saliva is commonly acid; and, in the severe and malignant diseases of this type, occurring in tropical climates, not only the saliva, but the whole assimilating organs, and even the blood itself circulating in these organs, have been observed to be in an acid condition.

" Now, the presence of so much lactic acid cannot be accounted for, except on the supposition that a certain portion of what ought to constitute, or actually has constituted, the albuminous, or, rather, gelatinous parts of the system, are decomposed or destroyed: and as gelatinous and albuminous matters or textures cannot be converted into lactic acid alone; that consequently, other unnatural and probably poisonous principles are developed in conjunction with the lactic acid; to which in part, as well as to the lactic acid, many of the secondary consequences of mal-assimilation are to be referred. In other words, the alimentary matters which ought to be converted into albumen, by the primary assimilating organs; and the albuminous matters of the blood, which, in the secondary assimilating processes, ought to be converted into the living gelatinous and albuminous tissues, are, by the deficient or disordered operations of the vital processes, converted, in a greater or less degree, into lactic acid, and other unnatural combinations." 82.

Dr. Prout next asks—what constitutes the difference between ague, rheumatism, and neuralgia, when the cause and general conditions of the system in these affections are assumed to be the same? And the only answer he can give is—difference in the degree in which the same organs are affected; or differences in the seat of disease or organs affected; or, what is most likely, a combination of both these kinds of differences. Difference in *degree* he thinks hardly competent to the effect. He is " driven to the conclusion, that these different forms of disease arise from derangements in the secondary assimilating processes proper to different tissues or structures. Thus we may suppose (and the supposition seems to be rendered probable by the phenomena) that, in intermittent fevers, the primary assimilating organs, the stomach, the liver, and the spleen, are principally in fault; that the secondary assimilating processes, by which the structure or tissue of these organs is produced and maintained, are impaired; and that to the consequent imperfect development of these organs we may not only refer the formation of the lactic acid, and other unnatural matters, generated during the digestive processes; but also those organic lesions and morbid hypertrophies, which are so apt to take place in the spleen, &c. during severe and long-protracted fevers of this type. In rheumatism, the same derangements, to a less extent, appear to exist in the primary assimilating organs; but, in this case, the secondary assimilating processes, by which the gelatinous portion of the muscular system and its appendages are produced and maintained, may be supposed to be more especially implicated; and the loss of power, and the great degree of pain usually present in rheumatism, may be referred to the disorder of the numerous nerves of motion and of sense, which, as well as the fibrinous portion of the muscles, are likewise necessarily affected by the derangements. Moreover, on these suppositions, we may explain the formation of the large quantities of lactic acid usually present in rheumatic affections, as well as the swelling, &c.; for as all the organs are more or less involved, and their functions paralysed, not only imperfect assimilation takes place in the part affected; but the apparatus destined to remove matters which are unfitted, or no longer useful, from the scene of operation, likewise cease to act; and hence such unfitted and useless matters accumulate, and cause swelling in the part affected. In simple neuralgic affections, nearly the same explanation may be given. Derangements of the primary assimilating processes, analogous to, or identical with, those existing in ague and rheumatism, are always present in a greater or less degree in these affections; while the derangements going on in those secondary assimilating processes, by which the nervous substance and its immediate appendages are produced and maintained, may be supposed to be the immediate cause of the pain and other distressing symptoms of the disease."

Only rheumatic neuralgia is here alluded to.

*Treatment.*—There are two classes of remedies—the empirical, consisting of quinine and tonics in general—and those counter-mechanical or chemical expedients, which are calculated to neutralise the effects of mal-assimilation.

In the treatment of the development of acidity during the primary assimilating processes, the first point to be determined, as far as we are able, is the nature of the *cause.* Then the empirical treatment may be applied. Thus, if the cause lies principally in the stomach itself, and the symptoms denote an inflammatory tendency, the due administration of local blood-letting, &c., will be found beneficial; if mere irritation be indicated, sedatives, as the hydrocyanic acid, various tonics, &c. will be found useful. If the cause be chiefly remote, as in the hepatic system, the employment of means calculated to remove inflammatory or passive congestion, as mercury and other deobstruents, will be indicated. If the cause be organic disease; and if such organic disease lie deep in the system, as in one of the great nervous centres, very little beyond palliatives can be advantageously employed. Frequently the cause is not isolated, and merely one of the three. They are complicated, and the case proportionably hard to manage.

The two great objects to be kept in view in the administration of the second class of remedies is either the mechanical object of getting rid of the unnatural material whose effects we wish to obviate; or the chemical object of neutralizing the acid, and other unnatural products of the primary assimilating processes. Now, as both these objects have reference to certain periods, and depend upon the *time when* the assimilating organs are called upon to perform their duty; it is obvious, that to obtain the utmost benefit of this class of remedies, their administration must in a great degree be regulated by such periods. Thus the acid residua of a meal should be neutralized when the digestive processes are completed; that is to say, from three to six hours after the meal has been taken; and for this purpose, even in the worst cases, from ten to twenty or thirty grains of the carbonate of potash will be quite sufficient. Four or five grains of nitre are a good addition to the carbonate of potash. This remedy must be steadily persisted in, till the affection has been entirely subdued by the joint effect of appropriate diet and medicines. For alkaline remedies *do not prevent acidity, but merely neutralize that formed.*

When acidity prevails in the lower portion of the intestinal canal, and particularly in the cœcum, the treatment must be modified to meet the circumstances. The soluble antacids in this case have comparatively little effect, from their being neutralized and absorbed before they reach the seat of the affection; hence the insoluble antacids, and particularly magnesia, will in general be found more useful in such cases. The shortest mode, however, of getting rid of the immediate inconvenience of acidity in the lower bowels, is usually to inject a pint or two of warm water, (or of soap and water,) and thus of removing the offending cause. Mild purgatives too are serviceable.

### GENERAL OBSERVATIONS ON THE PATHOLOGY OF ALBUMINOUS ASSIMILATION AND SECRETION.

The derangements of albuminous assimilation are best identified by the changes they induce in the urinary secretion. These changes form the basis of Dr. Prout's classification, which is as follows:—

*a.* Derangements of the assimilating processes, accompanied by excess or deficiency of urea in the urine.

*b.* Derangements of the assimilating processes, accompanied by the presence of albuminous matters in the urine.

*c.* Derangements of the assimilating processes, accompanied by the presence of lithic acid and its compounds in the urine; and,

*d.* Derangements of the assimilating processes, accompanied by the presence of cystic oxide in the urine.

### *Of an Excess and Deficiency of Urea in the Urine.*

Dr. Prout thinks these affections not sufficiently attended to.

*Of Affections connected with Excess of Urea in the Urine.*—The proportion of urea in healthy urine is such, that on the addition of nitric acid, no crystallisation takes place till the urine is concentrated by evaporation. In a variety of cases, however, the quantity of this principle is so increased, that crystallisation takes place on the addition of nitric acid, without any previous concentration of the urine; and in many such cases, on analysis, we find that this excess of the urea is not only absolute but relative; that is to say, that the quantity of urea in the urine is not only absolutely greater than natural, but relatively far greater to the other ingredients, than it is, or ought to be, in the healthy secretion. Now this absolute and relative excess of urea in the urine gives occasion to two forms or rather modifications of disease, which, as in diabetes, are chiefly distinguished by differences in the quantity of urine passed, viz. *Excess of urea without diuresis, and Excess of urea with diuresis.* These two forms of disease, precisely as in diabetes, without or with diuresis, sometimes gradually pass into each other in the same individual; and in fact they seem to differ from each other little more than in degree. In the first form of the disease, the quantity of urine passed seldom much exceeds the healthy standard, and in this case the quantity of urea is both absolutely and relatively greater than in health. In the second form of the disease, the quantity of urine is sometimes excessive; and in this instance the quantity of urea, in a given specimen of urine, may be less than in health; though the quantity of urea relatively to the other ingredients may be greater than natural; and the absolute quantity of urea passed in a given time, may thus, as in the other modification of the disease, exceed the natural standard.

In the first form of the disease, the average specific gravity of the urine seems to be a little above 1·020; and occasionally to vary from 1·015 to 1·030, or even higher. Most generally the secretion is transparent and pale coloured; but occasionally assumes somewhat the appearance of porter more or less diluted with water; and this variety in colour not unfrequently takes place in the urine of the same person. When first voided, the urine reddens litmus paper, and consequently has the usual acid reaction of healthy urine. Its only remarkable property is that of containing so much urea as to speedily form a crystallized compound on the addition of nitric acid. It is prone to decomposition, and soon becomes alkaline.

The patient has usually a frequent desire to make water, and the quantity voided in the twenty-four hours appears to be somewhat above the natural standard, and is influenced by slight causes. There is sometimes a sense of weight or dull pain in the back, accompanied by a disinclination to bodily exertion. The patient also complains of more or less uneasiness in the assimilating organs. The functions of the skin too appear to be little deranged; hence perspiration, from the fatigue it is apt to produce, often takes place readily under exercise.

In the second modification of the disease, in which the quantity of urine passed is excessive; besides most of the symptoms above enumerated in an aggravated form, there exists, in addition, more or less of thirst and morbid craving after food. The patient likewise complains of general coldness and great bodily weakness. In some instances also there is considerable emaciation; though not to the same remarkable extent as in diabetes.

*The causes* are allied to those which predispose to diabetes. Hereditary disposition is probably a common one. Abuse of the sexual powers in early life is another. Indiscretions in diet, anxiety, &c. are others.

Most of the subjects Dr. P. has seen have been middle-aged men, of thin and spare habit, with a sort of hollow-eyed anxiety of expression in their countenance; unusually nervous and susceptible, but by no means always hypochondriacs; and free also from gout, and, as far as could be ascertained, from structural disease of the urinary or any other organs.

Dr. Prout thinks the complaint rare—twenty, perhaps, of diabetes may be seen for one of it. But patients may pass it by until it has terminated in something worse.

The *proximate cause* of the disease may be found, in Dr. Prout's opinion, in derangements of the secondary assimilating processes, rather than of the primary; that is to say, that the chief source of the urea in the system is that peculiar modification of the albuminous principle distinguished as gelatine; and which, as is well known, is not found in the blood, nor in any previous stage of the assimilating processes; but is developed only during the secondary assimilating processes.

*Treatment.*—In the two first forms of the disease, the diet should be light and nutritious, but not stimulating; and in general should consist principally of animal and farinaceous matters. If the patient has been accustomed to the use of fermented liquors, a small quantity of the more generous wines, or sound porter, may be allowed; but all diluent and diuretic fluids should be abstained from as much as possible; and thirst should not be indulged.

Moderate exercise on foot and horseback, and abstinence from anxiety are to be enjoined.

No rough treatment of any sort should be attempted. Purgatives and alteratives may be required, but should be employed with caution. In both forms of the disease, and particularly in the second, sedatives are usually required, and of these opium is the chief. With the sedatives may be conjoined such tonics as seem to be suited to the individual habit; and as the complaint recedes, and the health becomes re-established, the sedatives may gradually be withdrawn.

*Of Affections generally connected with a Deficiency of Urea in the Urine.*

That the extrication of urea by the kidneys is indispensable is not only probable in itself, but accordant with experience, for Dr. P. has never found a specimen of urine, which, when recently passed, did not, on examination, prove to contain more or less of urea, or of its equivalent carbonate of ammonia. Yet there are several forms of disease, in which the proportion of urea is not only absolutely but relatively less than it is in healthy urine. A prominent symptom of these various affections is usually diuresis. The complaint varies somewhat in adults and children.

In adults, it may be considered under the heads of *Diuresis Intermittens* and *Diuresis Continua.* " Perhaps," says our author, " the nearest approach to an entire absence of urea in the urinary secretion, occurs in hysteria. Hysteric diuresis, however, is distinguished by being *occasional:* while, in the intervals, urine is often passed containing a greater proportion of urea than natural. Hysteric urine has often a specific gravity scarcely exceeding that of spring water; and as passed, is often limpid and colourless, and nearly free from sensible properties of every kind. When much concentrated, however, by evaporation, hysteric urine always, according to my observations, displays sensible colour and odour; and if examined in this condition, not only yields traces of saline matters, but of urea. Hysteric urine has sometimes a disagreeable odour when passed; and in almost all instances soon acquires a purid smell, like that of cabbage water; becomes more or less opake; and deposits crystals of the triple phosphate of magnesia and ammonia; especially in warm weather. Hysteric urine is not exclusively passed by females; but is occasionally voided by individuals of the other sex. In general the affection requires no specific treatment, but yields to appropriate remedies in common with the other symptoms of hysteria. Many nervous individuals, also, who cannot be said to be hysteric; or to be subject to any urinary disease, often, as is well known, pass large quantities of limpid urine on exposure to cold, and to various other exciting influences. Such urine generally differs from hysteric urine in being only *very dilute* healthy urine; while in hysteric urine the relative proportions of the ingredients are always deranged." The two, however, run into one another.

The diseases described by authors under the name of *Diabetes Insipidus*, are probably various in their nature. Generally they seem referable to the class under consideration. The urine voided in a given time is *constantly* much above the healthy standard; the patient usually drinking in proportion. It is almost as colourless as water; at other times it is of a very light straw colour; and its specific gravity in these instances is found to vary from the specific gravity of spring water, viz. 1·001 or 1·002, to 1·008 or 1·010. The more dilute specimens of urine are commonly quite neutral; but the heavier specimens have sometimes a faint acid reaction. The proportion of urea, as compared with that of the other ingredients, is usually less than natural; hence such urine, on being kept, often acquires a putrid or sour, rather than an ammoniacal smell. But so much urine is made, and this is so influenced by the fluids taken, that it varies greatly.

The constitutional symptoms vary too. But there is always great thirst; a dry state of the skin; and usually a constipated state of the bowels. In most cases, there is an uneasy sensation referable to the stomach, accompanied by a morbid craving for food; at other times this sensation merges into nausea, and there is a perfect indifference to solid matters; which are almost immediately ejected by vomiting. There are also more or less of emaciation; depression of spirits; and great muscular debility, with all their consequences.

*Causes.*—On this head Dr. Prout has little to say. However this form of diuresis may depend upon the nervous temperament, it certainly, in its turn, aggravates it. It seems to occur equally in both sexes, and is chiefly limited to the middle period of life. Sometimes, as already stated, it appears to be the natural consequence of the form of diuresis connected with an excess of urea. At other times it cannot be referred to any distinct cause. Dr. Prout believes that it is often connected with, or leads to, incipient disease of the kidneys; and if this opinion be correct, it may occasionally pass into the forms of disease marked by albuminous urine.

Diuresis, with deficiency of urea, in young children, is usually accompanied by symptoms very similar to those before-mentioned as occurring in diuresis with an excess of urea; but the symptoms are commonly much severer. The quality of urine is worse, being, often, more or less serous, and when the disease proves fatal, as it frequently does, the kidneys, as well as other organs, are found to be in a state of disease.

The diuresis of old people, and particularly of old men, is often accompanied by a deficiency of urea; but it is generally associated with some apparent organic disease, either of the kidneys or neck of the bladder, or both; to which, as causes, the diuresis appeared to have been chiefly referable. The urine in these cases is often alkalescent or slightly serous.

The complaint is often extremely obstinate, and when it appears to give way, is disposed to return from the slightest causes. It often, too, proves fatal from dropsical effusion or coma.

*Treatment.*—One of the first great principles to be attended to, is, as much as possible, to restrain the patient from drinking; for if he be allowed to drink *ad libitum*, it is in vain to hope for benefit from any plan of treatment. Another point to be kept in view, is to promote cutaneous action. For this purpose the vapour bath and friction, assisted by the internal use of Dover's powder, antimony, &c.; or, if the patient's circumstances admit, removal to a warm climate; will be found highly serviceable. Tonics of every kind usually disappoint our hopes; and the more active tonics especially, often increase the thirst. To these may be added *sound* porter, and diet consisting chiefly of animal and farinaceous matters. Active purgatives are mischievous.

The treatment of the disease in infants and young children is similar in principle to the treatment of diuresis accompanied by an excess of urea; but to ensure a chance of success, the remedies must be much more carefully and sedulously applied; and in spite of all that can be done, the complaint usually goes on to a fatal termination.

*Of Albuminous Urine.*

Dr. Prout remarks on the controversy which has existed and exists in reference to the organic lesions occasioning albuminous urine. But he takes no part in it. He adheres to his old opinions. He says—

"I considered the albuminous matters occurring in the urine as of two distinct kinds, viz. chylous and serous; in the first case, the albuminous matters of the urine were supposed to resemble the albuminous matters in the chyle; in the second case, the albuminous matters in the urine were supposed to be identical with the albuminous matters of the blood. I also remarked that distinctly defined instances of both these varieties of albuminous urine are rather uncommon; and that by far the most frequent form which the disease assumes, seems to be of a mixed character; that is to say, the albuminous matters in the urine partake more or less of both the chylous and serous characters." 112.

He therefore describes albuminous urine under the two heads of *chylo-serous urine* and *serous urine*, admitting that they run into each other, and that the latter is the more frequent.

*Of Chylo-serous Urine.*—We pass over, as our readers must be acquainted with it, the description of this sort of urine, and merely notice the conclusions drawn by Dr. Prout from thirteen cases of which he has seen more or less.

First. The disease occurs in both sexes before and after puberty. Of the thirteen cases, five were males and eight females. In three cases, two males and one female, the disease occurred before puberty; of these three cases, one was a male infant of about eighteen months old.

Secondly. Of the thirteen cases, seven occurred either in natives of hot climates, (East and West Indies,) or in individuals who had resided for many years in hot climates.

Thirdly. The general health suffers much less than might be expected. Two of the females, for instance, while labouring under the affection in a marked degree, became pregnant and brought forth healthy children. Hence the disease does not interfere with the generative functions; nor with the secretion or qualities of the milk.

Fourthly. Of the thirteen cases, three are known to be dead. Of the remainder, five, Dr. P. believes, are alive and well. Of the others he can give no account. In two of the fatal cases, the patients were cut off by acute attacks of inflammation of the abdominal viscera. The third patient, whose case will be subsequently given as an illustration, died apparently in a state of exhaustion and great emaciation, after labouring under the affection for nearly twenty years.

Fifthly. The disease is not necessarily connected with organic lesion of the kidney; at least organic lesion appreciable by the senses.

Sixthly. The causes of this affection, whether predisposing, exciting, or proximate, are imperfectly understood.

*Of Serous Urine.*—We shall merely, in the present article, *introduce* Dr. Prout's views on this important affection to our readers. In our next we shall complete our notice of the volume.

Dr. Prout observes that, strictly speaking, perhaps there are many varieties of disease belonging to this head, differing not only in degree, but even in kind from each other; but, in the present state of our acquaintance with these subjects, such varieties can with difficulty be distinguished. He considers, however, only two principal kinds or species, one of an *acute*, the other of a *chronic* character. The first of these species includes at least two varieties, differing from each other principally in degree; the second eight; and the whole ten varieties so gradually pass into each other, that it is not easy to define their exact limits. If there be, however, a distinguishing feature in the character of the urine between the acute and chronic species, it consists in the fact,—that in the two acute varieties, the urine on cooling frequently deposits the lithate of ammonia; while in four at least of the chronic varieties, this phenomenon never occurs under any circumstances.

Dr. Prout first defines the sense in which he understands and uses certain terms.

"The different meanings attached to the term *inflammation,* or what is the same thing, to words terminating in *itis,* by authors, are a frequent source of misconception, and consequently of mistakes, in medical reasonings. Many of the continental authors, for instance, and particularly the French authors, appear to consider inflammation as almost the only cause of disorganization; at least, it is difficult to arrive at any other conclusion, provided they employ the suffix *itis,* in its usual acceptation. Thus in the recent excellent work of M. Rayer, the diseases to be here and elsewhere considered, are arrangrd under the heads of *Nephritis, Pyelitis, Peri-nephritis,* &c. according as the secreting portion of the kidney, or the membranes lining its internal cavities, or covering its external surface, &c. are found, after death, to be affected with signs of recent inflammation. This view of the subject has always appeared to me to be imperfect. That inflammation is the *immediate* cause of death in most of these diseases is not denied; but admitting this, I ask, does the term inflammation, even when qualified by the epithet chronic or otherwise, rightly designate that comparatively quiescent state of the kidneys which immediately preceded the fatal inflammatory attack? The general answer to this question must, I think, be in the negative. That there is such a condition as chronic inflammation; and that such a state of chronic inflammation does, in some instances, exist in the kidneys previously to the more acute and fatal attack, is not doubted; but my decided opinion is, that in the greater number of instances, the previous state of the kidneys cannot by any justifiable latitudinarianism be designated by the term *chronic inflammation.*" 122.

All organic affections, he continues, may be supposed to arise from *degeneration* and *inflammation.* By degeneration, he means that slow and gradual change occurring in all living structures, which appears to be connected with, or to result from, the gradual decay of the vital processes in general, and particularly of the processes of assimilation. Degeneration, therefore, is the natural and universal consequence of age; but it may arise in early life from a variety of causes, among which the most frequent are: first, an *inherited* and *innate weakness* of the vital powers, either as they exist in the system generally, or as they exist in particular organs; as for instance in the kidneys; secondly, an *acquired* weakness of the vital powers in general, or as regards the vital powers of particular organs, produced by a variety of slowly acting causes; such as continued errors in eating and drinking; long exposure to the influence of unhealthy situations; or of occupations unfavourable to the general health, &c.; and, thirdly, an *acquired* weakness of the vital powers, either general or local, produced by the operation of acute causes; as acute inflammation, severe accidents, &c.

The term *inflammation* he employs in its admitted sense, and considers three grades or kinds of it; viz., *acute* inflammation, or inflammation in its most active form; such as it more especially exists in healthy subjects and in healthy organs: *chronic* inflammation, or that obscure state of activity, which, for want of a better term, we designate inflammation; and which is almost exclusively limited to degenerated structures: and *congestive* or *adynamic* inflammation, such as occasionally follows acute inflammation in healthy subjects; but much more generally takes places in unhealthy subjects; or succeeds to the chronic inflammation of degenerated structures.

He deduces the following inferences from these views:—

First. A degenerated condition of an organ, from whatever cause produced, may exist for a greater or less period in a state of comparative quiescence; during which state of quiescence, the system in general may accommodate itself more or less perfectly to the degenerated state of such organ.

Secondly. Local degenerations are liable to become aggravated from a variety of causes, and particularly from inflammation; and when such causes have ceased to operate, as for instance when the inflammation has subsided; the general system, as before, gradually accommodates itself, during the succeeding period of quiescence, to the new order of things induced by the inflammatory attack.

Thirdly. Such alternations of comparative quiescence and of activity repeatedly take place; the degeneration on the whole being increased during each successive paroxysm; till finally the patient is cut off during an inflammatory attack, which overwhelms his exhausted powers.

Fourthly. The patient in such cases cannot be said to die of inflammation alone; but of the conjoint effects of degeneration and of inflammation. Moreover, the inflammation would probably never have taken place, had degeneration not existed as a predisposing cause: or having taken place in a perfectly healthy structure, the inflammation would not have proved fatal.

Fifthly. The appearances presented after death under these circumstances are often very unsatisfactory, and quite useless in a pathological point of view; inasmuch as by presenting the conjoint effects of degeneration and of inflammation, they do not enable us to distinguish what is due to degeneration, and what to inflammation; a distinction in all instances of the utmost practical importance.

Reverting to the kidney, Dr. Prout considers the *serous* character of the urine with reference to the kidney in a state of *health* (sp. *a.*); and with reference to the kidney in a state of *degeneration* (sp. *b.*); and as further *varied* by the accidental circumstances of *quiescence*, and of *inflammation*. So that the arrangement stands thus:—

| | |
|---|---|
| Species *a. Serous Urine; the Kidney in a State of Health.* | *var.* 1 *Quiescent.* |
| | *var.* 2 *Inflamed.* |
| Species *b. Serous Urine; the Kidney in a State of Degeneration.* | *var.* 1 *Quiescent.* |
| | *var.* 2 *Inflamed.* |

In treating of degenerated kidneys, Dr. Prout employs the terms *anæmotrophy* and *hæmotrophy* to imply a-deficiency and an excess of sanguineous nourishment. "*Atrophy* and *hypertrophy*," says he, " as commonly understood, include the idea of diminished and increased magnitude; and do not, therefore, exactly express the meaning intended to be conveyed. On the other hand, *anæmia* and *hyperæmia* have reference only to the quantity of blood present, without regard to its nutritive properties. For these reasons, as well as for the sake of distinction, I have adopted the terms in the text to designate those peculiar conditions of degenerated organs, chiefly characterized by the absence or presence of (red) blood; and which conditions apparently depend on the individual contraction or expansion, or on the numerical diminution or increase (or both) of the blood-vessels supplying such degenerated parts. That some such distinction is requisite for describing the condition of degenerated structures, is evident from the fact, that an organ may be anæmotrophied or hæmotrophied, without being diminished or increased in bulk; or without the presence of general anæmia or hyperæmia; and *vice versâ*."

And in reference to this anæmotrophic and hæmotrophic condition of the kidney, he thus classifies the affections of it in a state *degeneration*:

| Species | Section | Subspecies | Varieties |
|---|---|---|---|
| Species *b. Kidney Degenerated.* | Sect. i. In a State of Anæmotrophy. | Subspecies α. The kidney in a state of *organic change*; but without any *visible* derangement of its ultimate structure. | *Var.* 1, *Quiescent.* *Var.* 2. *Inflamed.* |
| | | Subspecies β. The kidney in a state of *disorganization*, i. e. having its ultimate structure more or less visibly destroyed. | *Var.* 3. *Quiescent.* *Var.* 4. *Inflamed.* |
| | Sect. ii. In a State of Hæmotrophy. | Subspecies γ. The kidney in a state of *organic change*; but without any *visible* derangement of its ultimate structure. | *Var.* 5. *Quiescent.* *Var.* 6. *Inflamed.* |
| | | Subspecies δ. The kidney in a state of *disorganization*, i. e. having its ultimate structure more or less visibly destroyed. | *Var.* 7. *Quiescent.* *Var.* 8. *Inflamed.* |

Here we pause for the present. As we before observed, we shall return to the work in our next Number. And a very remarkable work it is, destined, if we mistake not, to effect material changes in the science of medicine, and to exercise a not inconsiderable influence upon its practice. Whatever we may think of the extent to which Dr. Prout has carried his views, none can refuse to him the merit of a profound thinker and a most sagacious observer. His views are perhaps the boldest and most original of our time, and their philosophic character is a record at once of the knowledge of the day and the genius of their author. It would be wrong to conceal our humble opinion that something of speculation is mixed up in them, and that they occasionally border on the fanciful. The chemist, likewise, is somewhat too apparent, and the humoralism, though of the most exalted and scientific character, may be deemed a little too prominent. But so masculine a mind as Dr. Prout's, possessed of a mode of analysis so powerful, may be well excused for applying it too widely, and for attempting to reduce too numerous phenomena within its sphere. This is a slight error which will bring its own correction,

Our readers, we are sure, will not be displeased at our lengthened account of a volume which teems with such valuable facts and equally valuable reasoning. In our next, that account will be completed.

